# Design of graphenic nanocomposites containing chitosan and polyethylene glycol for spinal cord injury improvement

**DOI:** 10.1039/d1ra00861g

**Published:** 2021-06-04

**Authors:** Ayda Yari-Ilkhchi, Abbas Ebrahimi-Kalan, Mehdi Farhoudi, Mehrdad Mahkam

**Affiliations:** Chemistry Department, Faculty of Science, Azarbaijan Shahid Madani University Tabriz Iran mmahkam@yahoo.com mmahkam@gmail.com mahkam@azaruniv.ac.ir +98 4134327541; Faculty of Advanced Medical Science, Tabriz University of Medical Sciences Tabriz Iran; Neurosciences Research Center (NSRC), Tabriz University of Medical Science Tabriz Iran

## Abstract

Advanced therapeutic strategies include the incorporation of biomaterials, which has been identified as an effective method in treating unsolved diseases, such as spinal cord injury. During the acute phase, cascade responses involving cystic cavitation, fibrous glial scar formation, and myelin-associated dissuasive accumulation occur in the microenvironment of the spinal cord lesion. Graphene oxide (GO)-based materials, due to their extraordinary chemical, electrical and mechanical properties and easy to modify structure, are considered as rising stars in biomaterial and tissue engineering. In order to enhance the biodegradability and biocompatibility of GO, cell proliferation may be appropriately designed and situated at the lesion site. In this study, chitosan (CS) and polyethylene glycol (PEG) were grafted onto GO sheets. CS is a natural non-toxic polymer with good solubility and high biocompatible potential that has been used as an anti-inflammatory and anti-oxidant agent. Furthermore, PEG, a synthetic neuroprotective polymer, was used to develop the pharmacokinetic activity and reduce the toxicity of GO. Herein we report a novel nanocomposite consisting of PEG and CS with a potential advantage in spinal tissue regeneration. The preliminary *in vitro* study on mesenchymal stem cells (MSCs) has demonstrated that the prepared nanocomposites are not only non-toxic but also increase (by nearly 10%) cell growth. Finally, the use of mixed nanocomposites in the spinal cord injury (SCI) model resulted in good repair and inflammation decline after two weeks, such that walking and functional recovery scores of the hind limbs of mice were improved by an average of 6 points in the treatment group.

## Abstract 2

Herein we report a novel nanocomposite consisting of PEG and CS with a potential advantage in spinal tissue regeneration.

## Graphical abstract

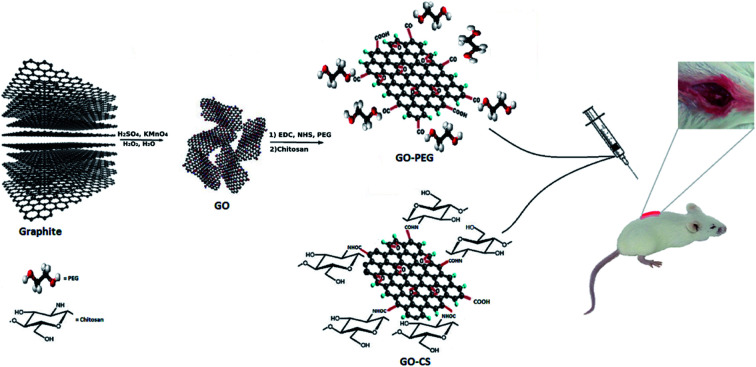

## Introduction

Due to the physical anatomy of the nervous system (NS), the reconstruction and the total treatment of injured nerves have been proved to be more delicate compared to the treatment of other tissues. The human NS consists of the central nervous system (CNS) including the brain and spinal cord and the peripheral nervous system (PNS).^1^ Annually, three main factors, namely road traffic accidents, falls and stricture, cause partial to total physical damage and even death. Due to increasing awareness of SCI mechanisms, the treatments available to these patients tend to stay popular.^2–4^ Traumatic injury to the human spinal cord causes irreparable damage, producing a condition that prevents damaged neurite regrowth.^5^ Studies showed that damaged nerve cells would regenerate and permit partial original nerve conduction with sufficient therapeutic interference.^6^ Appropriate biomaterial structures that are embedded in the damaged spinal cord may enhance the microenvironment, facilitate SCI improvement and provide a solution to SCI challenges.^7–9^

GO and its derivatives have been widely studied in many different fields since its invention in 2004.^10^ Its excellent electrical,^11,12^ optical,^12^ magnetic,^13^ thermal and mechanical properties^14^ result in the wide ranging application of these nanomaterials in neuroscience,^15,16^ biomedicine,^17,18^ bioimaging,^19,20^ manufacture of biosensors,^21^ drug/gene delivery,^22^ phototherapy^23^ and tissue engineering.^24–26^

Some research groups have recently investigated graphene and its toxicity in cells and animals. They have shown that numerous factors such as concentration, size, lateral dimension, surface chemistry, and aggregation status have an effect on toxicity. Physical adsorption of graphene, with its sharp corners, results in penetration of cell membranes, causing serious harm to the membrane and leakage of cytoplasmic content. Pristine graphene induces macrophage apoptosis by decreasing the potential of the mitochondrial membrane and increasing reactive oxygen species (ROS).^25,27–29^

Numerous techniques have been developed to overcome these drawbacks of pristine GO, capable of promoting the production of new GO derivatives with high biodegradable and biocompatible properties, encouraging cell adhesion, and differentiating and imitating the cells’ natural environment. CS is a natural, linear polysaccharide extracted by partial chitin deacetylation. The primary amino group on CS forms H-bonds with the epoxy and carboxylic acid groups on GO, consequentially imparting unique characteristics such as biocompatibility, antibacterial behavior, and mucoadhesive properties. This reduces toxicity, improves delivery efficiency and helps to repair the damaged tissue.^30–33^

PEG is a synthetic, biocompatible and water-soluble polyether of various molecular weights, which binds to GO surfaces covalently and non-covalently.^34^ PEG attachment enhances the stability, water-solubility and absorption of GO, reduces its overall toxicity in physiological solutions and improves its drug/gene delivery and pharmacokinetic behaviour.^35–37^ Investigations of the efficacy of PEG in SCI demonstrated activities such as inflammatory response inhibition, neuroprotection, suppression of changes in the SCI microenvironment, and passing the blood–brain barrier or blood–spinal cord barrier which limits ROS.^38^

In this study, GO was proved to have neuroprotective and neuro-recovery effects. Indeed, GO-based nanomaterials with excellent properties provide an effective platform for neural regeneration. Here, grafting CS and PEG onto GO sheets is proposed to effectively improve their desired properties, such as reducing toxicity by increasing biocompatibility and biodegradability, and boosting solubility and antibacterial activity, to create a novel and suitable platform for nerve tissues.

In the present study, GO was synthesized through the modified Hummers method and its surface was modified by CS and PEG grafting, then the structural characteristics were determined *via* FTIR, XRD, TGA, DSC, SEM, DLS and zeta potential measurements. By investigating the toxicity of GO, GO–CS and GO–PEG towards mesenchymal stem cells, and SCI model recovery, we expect to furnish profound knowledge of the interactions between graphene oxide derivatives in living cells and their improvement effects on the damaged spinal cord.

## Experimental section

### Materials

Natural flake graphite, potassium permanganate (KMnO_4_), sodium nitrate (NaNO_3_), concentrated sulfuric acid (H_2_SO_4_), hydrogen peroxide (H_2_O_2_, 30%), hydrochloric acid (HCl), acetic acid (CH_3_COOH, 99.9%), dimethyl sulfoxide (DMSO), sodium hydroxide (NaOH) and chloroacetic acid (CH_2_ClCOOH) were purchased from Merck, and chitosan (medium molecular weight), polyethylene glycol 400, 3-(4,5-dimethylthiazol-2-yl)-2,5-diphenyltetrazolium bromide (MTT), *N*-(3-dimethylaminopropyl)-*N*′-ethyl carbodiimide hydrochloride (EDC) and *N*-hydroxysuccinimide (NHS) were purchased from Sigma-Aldrich. Dulbecco’s modified Eagle medium (DMEM), fetal bovine serum (FBS), penicillin–streptomycin, trypsin and phosphate-buffered saline (PBS) were obtained from Gibco.

### Measurements

The chemical composition and the structure of the nanocomposites were characterized by Fourier transform infrared (FTIR) spectroscopy, X-ray diffraction (XRD), thermogravimetric analysis (TGA), differential scanning calorimetry (DSC), dynamic light scattering (DLS) and scanning electron microscopy (SEM). Scanning electron microscopy (MIRA3 FEG-SEM) was used to distinguish the morphology of the pure graphite, GO, GO–CS and GO–PEG surfaces. After tempering of the specimen on the metal rim, a thin layer of gold under vacuum was sputtered onto the surface to prepare it for examination. To study the conjugation and composition, Fourier transform infrared (FTIR) spectra were recorded using a Bruker Vector-22 FTIR spectrometer in the range between 4000 and 400 cm^−1^. 1 mg of sample (graphite, GO, CS, GO–CS, PEG and GO–PEG) was mixed with 4 mg of KBr and compressed manually to prepare the pellets. The recorded spectra are displayed as wavenumber (cm^−1^) *vs.* percentage transmittance. Powder X-ray patterns were acquired from dried nanocomposite samples on a Bruker AXS model D8 Advance diffractometer using CuKα radiation (*λ* = 1.542 Å), with the Bragg angle ranging from 2 to 70° at 40 kV and 25 °C. For thermogravimetric analysis (TGA), samples were weighed and analyzed under a nitrogen atmosphere, at temperatures of 50–750 °C with a heating rate of 10 °C min^−1^, using a Q500 (TGA Q500) analyzer. Differential scanning calorimetry (DSC) was performed (DSC 822 from Mettler-Toledo, Switzerland) in the temperature range of 40–350 °C at a heating rate of 10 °C min^−1^ under nitrogen purge. In order to determine the average size, surface charge and polydispersity index (PDI) of the synthesized graphene derivatives, samples were dispersed in DW and were evaluated using DLS (Microtrac, Nanotrac Wave) at room temperature.

### Preparation of GO

GO was prepared through a modified Hummers method.^39^ Briefly, graphite (1 g) was mixed with concentrated H_2_SO_4_ (23 mL), followed by the addition of NaNO_3_ (0.5 g) at 0 °C overnight to exfoliate the graphite layers. The color changed to dark green upon gradual addition of KMnO_4_ (3 g) to the solution in an ice bath (due to the exothermic reaction) and stirring for 2 h. The mixture was then stirred for 1 hour at 40 °C, and its color changed to brown. Subsequently, excess deionized water (DW) was added dropwise to the above mixture and the temperature was then increased to 95 °C. The reaction was completed by adding 10 mL 30% H_2_O_2_ aqueous solution to the deep brown mixture until the mixture color changed to brilliant yellow. The resulting suspension was centrifuged at 12 000 rpm for 5 minutes, washed with 5% HCl and deionized water to neutralise the solution, and then dried at 60 °C for 6 h.

### Preparation of GO–CS nanocomposites

0.2 g GO powder was dispersed into 100 mL of DW followed by sonication for 1 h to form a yellowish-brown homogeneous suspension. 5 mL CS solution (1 wt%) in acetic acid was added and sonicated for another 30 min, then stirred at 40 °C for 3 h. The color of the mixture changed to black, and then the mixture was allowed to cool to room temperature. To remove unreacted CS, the mixture was washed twice with 0.1 M acetic acid solution and neutralized using dilute NaOH and distilled water. Finally, the prepared solid was dispersed again and dialyzed (molecular weight cut-off 14 kDa) with DW for three days at room temperature.

### Preparation of GO–PEG

Firstly, a GO suspension (1 mg mL^−1^) in DW was dispersed in a sonication bath for 2 h. Afterward, 1.2 g sodium hydroxide (NaOH) solution and 1.0 g chloroacetic acid (CH_2_ClCOOH) were added into the GO suspension, dispersed for another 30 min and collected. All of the collected activated GO sheets were re-dispersed in DW and mixed with 10 mg of PEG. After adding EDC (4 mg) and NHS (6 mg), the homogeneous mixture was stirred vigorously at 40 °C overnight. During the reaction, the yellowish-brown aqueous solution changed to black. Subsequently, to remove the excess PEG, the resulting product was washed with DW five times and centrifuged. The obtained solid was dried at 40 °C before further use.

Before use in cells and the SCI model, all samples were washed with 70% ethanol, dried under a sterile hood and sterilized with UV irradiation for 30 minutes.

### Isolation and culture of MSCs

MSCs were isolated from the bone marrow from the femur and tibia bones of 2.5 month-old mice, as mentioned previously.^40^ In brief, the whole body of the animal was soaked in 70% (v/v) ethanol and dissected. Muscles, ligaments and tendons were gently detached from the tibias and femurs using micro dissecting scissors and a surgical scalpel. The bones were transferred to sterile PBS on ice. In a sterile laminar flow hood, the marrow cavity ends of the bones were cut and slowly flushed with DMEM until the bones became pale and centrifuged at 2500 rpm for 5 minutes. The supernatant was discarded, 5 mL complete DMEM (containing DMEM, 10% FBS, 100 U mL^−1^ penicillin and 100 mg mL^−1^ streptomycin) was added, and the mixture was transferred into a cell culture flask. The dish was incubated at 37 °C in a humidified atmosphere containing 5% CO_2_ to obtain 70–90% confluence. To obtain pure MSCs (removing blood cells, macrophages and fats), the extracted cells were passaged three times every 6 days and characterized by CD34, CD44 and CD90 surface markers through the flow cytometry method.^40^

### *In vitro* cell viability and cytotoxicity

The cytotoxicity and biocompatibility of the synthetic nanocomposites were determined using an MTT assay. First, 5000 cells per well were seeded in 96-well plates and incubated overnight to attach the cells to the dishes. The cells were then treated with increasing concentrations (0.1, 1, 10, 20, 40, 100, 150 μg mL^−1^) of GO, GO–CS and GO–PEG. After incubation at 37 °C for 24, 48 and 72 h, the 96 wells were washed with PBS and incubated with a fresh culture medium containing MTT (0.4 mg mL^−1^ medium) at 37 °C for 4 h in darkness. Afterward, to remove the unreacted dye, DMSO was applied to dissolve the intracellular, insoluble violet formazan substance to form a colored solution. The optical density (OD) of the formazan solution was read utilizing a spectrophotometric plate reader (Multiskan MK3, Thermo Electron Corporation) at 570 nm wavelength, and the outcomes were expressed as OD after blank subtraction.Cell viability (%) = OD_(test)_/OD_(control)_

### Modeling of SCI in animals

All the animal experimental protocols and processes were performed in accordance with the Guidelines for Care and Use of the Faculty of Advanced Medical Sciences of Tabriz University and approved (214/D/21085) by the Institutional Animal Ethics and Use Committee of Azarbaijan Shahid Madani University. All of the BALB/c mice used in this research were housed under standard laboratory conditions (humidity, 22–23 °C and 12/12 dark/light cycles). Mice (3 male + 1 female) were randomly assigned to the control (SCI) and treatment (SCI + COM) groups. The animals were anesthetized with ketamine + xylazine (80 : 20 mg kg^−1^). Laminectomy was performed at the T10 level and injury was induced by the compression method (0.3 mm, 15 s).^41^ After the damage, 50 μL nanocomposite complex, which consisted of GO–PEG and GO–CS (50 : 50 μg mL^−1^), was instantly injected into the lesion cavity and the wound was sutured. After surgery, urine was removed from the bladder until the mice could urinate naturally, and the mice received constant amounts of antibiotics and sterile normal saline for 5 days.

### BBB tests

The Basso, Beattie, and Bresnahan (BBB) test, a 21-point locomotor rating scale, was used to determine the locomotor action of the hind limbs after SCI. According to the BBB test,^42^ the evaluation was carried out daily for two weeks by two unrelated observers.

### Histological examination

Two weeks after nanocomposite treatment, the mice were anesthetized with an intraperitoneal injection of ketamine (80 mg kg^−1^) and xylazine (20 mg kg^−1^), and then the heart was perfused with approximately 20 mL of sterile normal saline solution to remove the blood and 20 mL 4% paraformaldehyde to fix the organs. After quick removal of the vertebral column, dorsal laminectomy was accomplished along the vertebral column’s length around the injury site to uncover the spinal cord. The spinal cord was carefully removed from the vertebrae and immersed in 4% paraformaldehyde solution. Fixed spinal cords were embedded in paraffin and cut into 5 μm-thick segments. After staining by hematoxylin and eosin (H&E), the anatomical features were examined with an optical microscope (Nikon Eclipse E100).

### Statistical analysis

Quantitative analyses were conducted using Graph Pad Prism and ImageJ Fiji. Data set means were calculated using the Student’s *t*-test and one-way ANOVA analysis for two or more experiments. The differences between the groups were found to be statistically significant (*p* values < 0.05 and *p* < 0.0001).

## Results and discussion

**Fig. 1:**
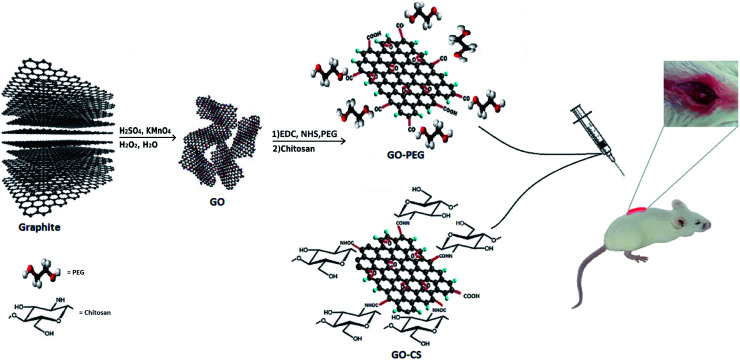
Schematic illustration of the procedure.

GO was synthesized *via* a modified Hummers method, in which graphite was intercalated with sulfuric acid and oxidized with KMnO_4_ to obtain epoxy, carboxylic acid and hydroxyl groups on its surface. Using extra potassium permanganate increased oxidation.^43^ In order to improve the medical activity of GO, its surface was modified with medium molecular weight chitosan and PEG through a self-assembly reaction in aqueous solution by way of H-bonding and electrostatic interactions. Different measurements confirmed successful conjugation onto GO. A comprehensive overview of the processes in this research is displayed in Fig. 1.

**Fig. 2:**
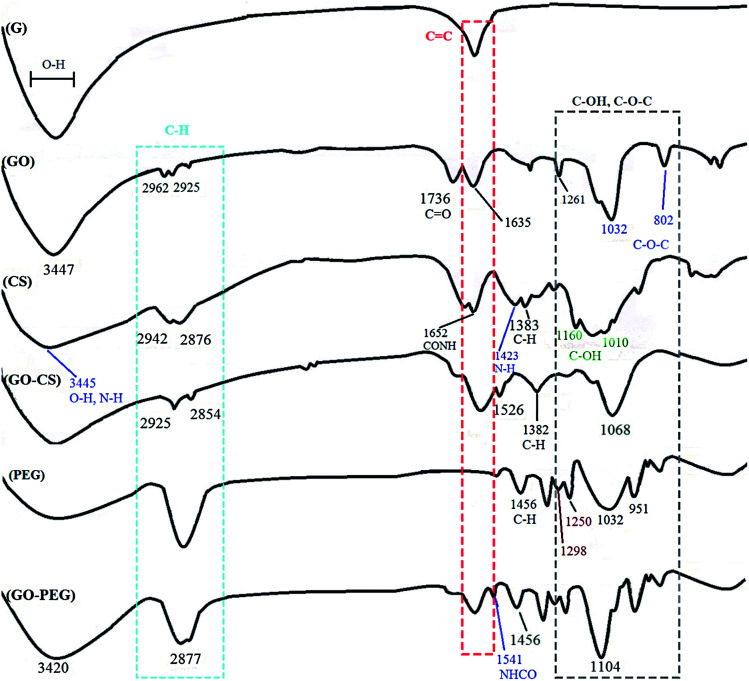
FTIR spectra of G, GO, CS, GO–CS, PEG and GO–PEG.

FTIR is an appropriate technique to monitor the synthesis and functionalization of GO. According to Fig. 2, although the graphite spectrum has no significant peaks, various groups of peaks were observed following the oxidation reaction to form GO. The strong band at 3447 cm^−1^ was due to –OH groups. The C–H stretching bands were observed at 2925 and 2962 cm^−1^. The characteristic C=O peak of carboxylic acid groups at 1736 cm^−1^, the C=C peak of conjugated ketones at 1635 cm^−1^ and the C–O stretches of epoxy groups and primary alcohols at 1032 and 1261 cm^−1^ were observed.^44^ For chitosan, the peaks at 3445 and 1423 cm^−1^ are due to NH_2_ stretching and bending vibrations, respectively, the peaks at 2876 and 1383 cm^−1^ are assigned to C–H, and the peaks at 1010 and 1160 cm^−1^ correspond to the primary alcoholic group C_6_–OH and the secondary alcoholic group C_3_–OH, respectively.^45^ The absorption peak at 1652 cm^−1^ is due to the carbonyl stretching vibration of the amide group. The characteristic absorption peaks of CS and GO–CS approximately overlap with each other.^46^ In the GO–CS FTIR spectrum, the presence of the NHCO stretching vibration at 1623 cm^−1^ is because of the reaction of the chitosan NH_2_ groups with the carboxylic acid groups of GO and the formation of an amide linkage. The carboxylic acid peak at 1736 cm^−1^ disappeared when compared with GO, showing that the NH_2_ groups in CS interacted with the carboxylic groups to build an amide linkage. Also, the epoxy/primary alcohol C–O stretch of GO at 1032 cm^−1^ was stronger and shifted to 1068 cm^−1^, owing to the interaction with the OH groups of CS. The characteristic secondary amide (N–H bending) signal shifts from 1600 cm^−1^ in the CS spectrum to 1526 cm^−1^ in the GO–CS spectrum, showing the presence of newly developed amide bonds between GO and CS. In the spectrum of GO–PEG, due to the interactions of the OH groups of PEG with the carboxylic acid groups of GO, the C–O stretch observed at 1032 cm^−1^ for GO occurred at extra high energy and shifted to 1104 cm^−1^. The band at 1541 cm^−1^ was attributed to the vibration of amide functional groups. The peaks at 2877 cm^−1^ and 1456 cm^−1^ were assigned to stretching and bending vibration of the –CH_2_ (sp^3^) groups of PEG, respectively. Meanwhile the peaks at 1250 cm^−1^ and 1298 cm^−1^ correspond to the bending vibrations of C–OH groups, indicating that PEG has been grafted onto GO–PEG.^47,48^

**Fig. 3:**
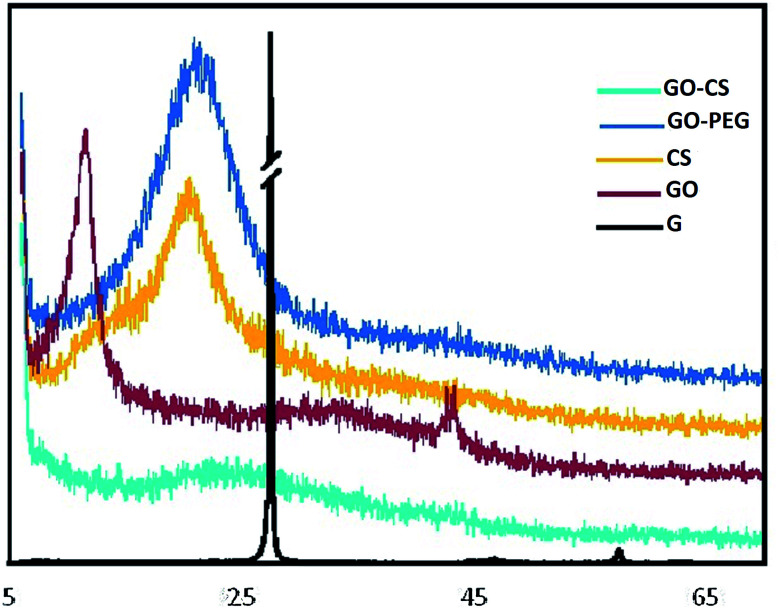
XRD diffractograms of G, GO, CS, GO–CS and GO–PEG.

XRD further confirmed the FTIR data. The XRD patterns of graphite, GO, CS, GO–CS and GO–PEG composites are depicted in Fig. 3. As shown by the patterns, natural graphite shows a sharp peak near 2*θ* = 26.6° (002) with an interlayer spacing of *d*_002_ = 3.35 Å; this peak exhibits a high degree of crystallinity.^49^ In the diffractogram of GO, the graphite peak changes to a wider and shorter one at 2*θ* = 11.6° due to the oxidation, and the (002) planes are shifted to (001). Due to the presence of polar groups between graphite layers, the interlayer distance increased obviously in a range from 3.35 Å to 8.1 Å.^50^ The CS pattern displayed a broad peak at 2*θ* = 20.74° (100); after the addition of GO (GO–CS), the diffraction intensity of CS obviously decreased and a broad peak centered at 2*θ* = 23.48° was observed, implying an amorphous structure and effective intercalation of CS chains between GO layers.^51^ The characteristic diffraction peaks of PEG appear at 2*θ* = 19.2°, 23.3°, 26.2°, 26.9° and 29.2°.^52^ The GO–PEG pattern exhibited a wide peak at 2*θ* = 21.44° (120) with interlayer spacing of 4.26 Å. Meanwhile the diffraction intensity of the characteristic GO peak at 2*θ* = 11.6° declined and the peak shifted to 2*θ* = 6.3°; the interlayer distance significantly increased to *d* = 14.1 Å, indicating the successful exfoliation of GO plates and the distribution of PEG into the interlayer spacing of GO. The broad (120) peak at 2*θ* = 21.44° corresponds to the crystalline structure of GO–PEG.

**Fig. 4:**
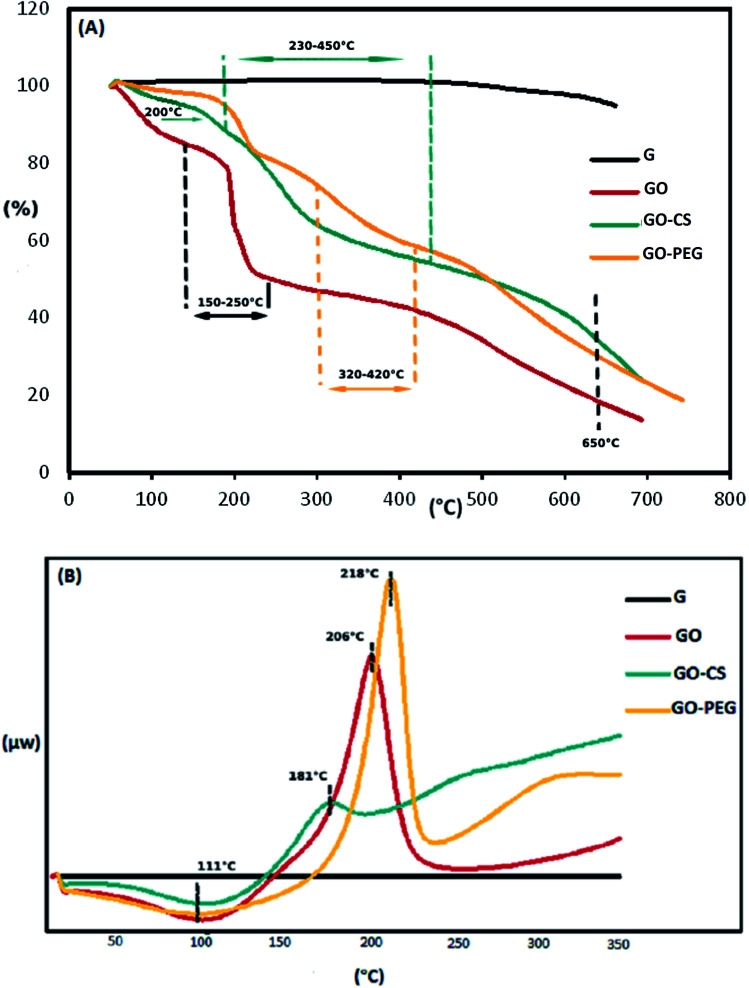
TGA (A) and DSC (B) diagrams of G, GO, GO–CS and GO–PEG.

The weight loss of the materials as a function of temperature was investigated by TGA, at a heating rate of 10 °C min^−1^ in a nitrogen environment. The TGA profile in Fig. 4A indicates that the GO sheets underwent a remarkable (25.1 percent) weight loss at 150–250 °C, resulting from pyrolysis of the oxygen-containing functional groups on the surface of the GO. The initial weight loss of the samples near 100 °C and the weight loss at 300–650 °C may be respectively attributed to the evaporation of absorbed water and the decomposition of the graphitic content. Obviously, the mass loss of the GO–CS composite is lower than that of GO, particularly the mass decline at 200 °C (∼8 percent), which shows a gradual decrease in the amount of oxygen-containing functional groups. But then, greater mass losses were observed in GO–CS at 230–450 °C (34%) due to CS decomposition.^50^ Three stages of thermal decomposition were perceived for GO–PEG. The mass loss between 320 and 420 °C could result from the decomposition of grafted PEG.^50^ The fact that functionalization of graphene oxide with CS and PEG results in superb stability is related to the strong connections between the organic units in GO and CS or PEG.

The TGA results were verified by the DSC curves. The related DSC findings (Fig. 4B) reveal no variation for graphite in the temperature range from 40 to 350 °C due to the high crystal content of carbon layers. Besides that, GO exhibits a wide endothermic peak at 50–150 °C, because of the exclusion of absorbed water molecules, and a sharp exothermic peak at 160–240 °C due to decomposition of oxygen-containing groups.^53^ This exothermic peak is much weaker in the DSC curve of GO–CS (170–200 °C), but there is another broad exothermic peak above 230 °C, indicating that GO–CS is more thermally stable than GO. Meanwhile, the exothermic peak of GO reduction in the DSC thermogram of GO–PEG was sharper and moved to a higher temperature (218 °C). The electrostatic forces and hydrogen bonds between PEG chains and GO sheets improve the thermal stability of GO–PEG. Because of the normal difficulties with the DSC technique, such as lack of accuracy, DSC analysis was not effective in detecting the glass transition temperatures (*T*_g_) of the composites.

**Fig. 5:**
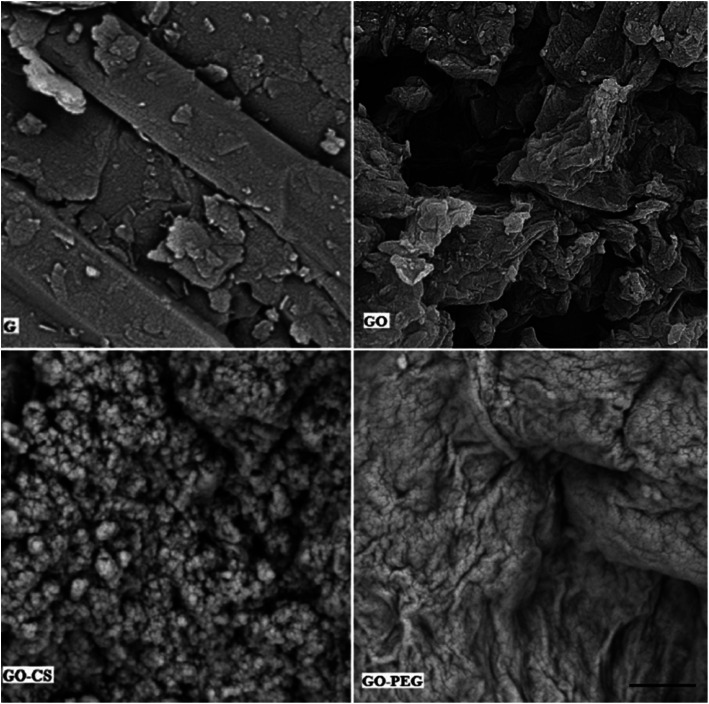
SEM images of G, GO, GO–CS and GO–PEG. The scale bar is 2 μm.

Subsequent evidence for this assessment is provided by morphological analysis. The SEM micrographs in Fig. 5 confirmed the larger and layered surface of GO as compared to that of GO–CS. The sponge-like GO–CS planes may be attributed to the non-covalent physical adsorption of chitosan on both sides of the GO sheets. GO–PEG exhibits a very wide, flat and aggregated structure. The PEG polymers are located between two-dimensional GO sheets and interact by H-bonding with both GO surfaces, clearly causing a reduction in interlayer space and a new three-dimensional network. This phenomenon is in line with the results of the previously described XRD analyses.

After preparation, the nanostructures must be characterised to ensure that they are suitable for medical applications, both “*in vitro*” and “*in vivo*”. According to the IUPAC recommendation, the PDI is a measure of the distribution of molecular weight and defines the heterogeneity index. As shown by the DLS results in Table 1, the PDI values of GO–CS and GO–PEG are in the range (0.05–0.7) defined by ISO standard documents 13321:1996 E and ISO 22412:2008.^54^ After attachment to CS and PEG, the average colloidal size of GO (170 nm) increased to 220 nm and 600 nm, respectively, however the PDI decreased due to the ionic interactions between GO and the modifiers. The successful synthesis of GO–CS was further confirmed by zeta potential analysis. GO had a zeta potential of −44 mV and GO–PEG had a zeta potential of −9 mV. Moreover, GO–CS was positively charged with a zeta potential of +32 mV after functionalization with cationic CS. Positively charged GO–CS can actively interact with negatively charged cell membranes, resulting in increased cellular uptake.

**Table 1:** Physicochemical characteristics of GO, GO–CS and GO–PEG

	GO	GO–CS	GO–PEG
Mean size (nm)	∼170	∼220	∼600
Zeta potential (mV)	−44	+32	−9
PDI	0.63	0.32	0.41

**Fig. 6:**
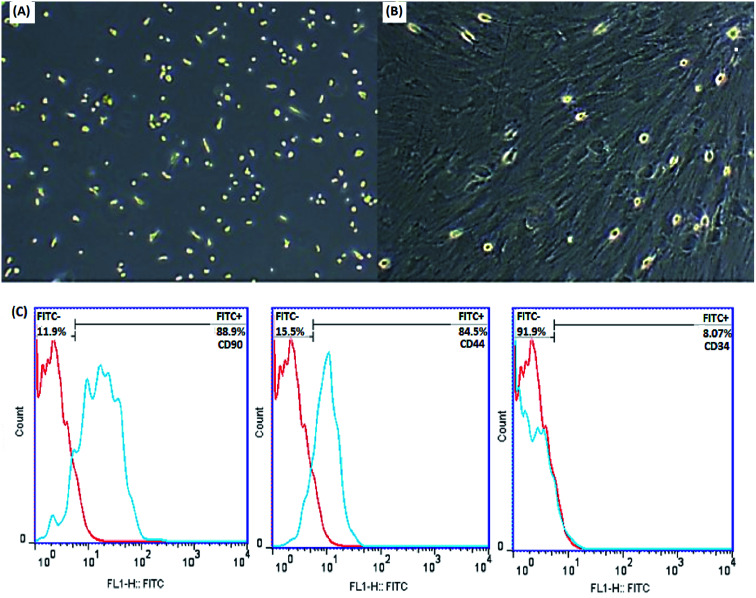
Identification of MSCs. Morphological characterization of MSCs by an inverted microscope after extraction (A) and after the third passage (B) (original magnification 100×), (C) flow cytometric analysis of MSCs after the third passage showed positive expression for CD90 and CD44 and negative expression for CD34.

MSCs are derived from mouse bone marrow and possess the capacity to self-renovate and differentiate into different cell species such as chondrocytes, osteoblasts, adipocytes and neurons due to their multipotent properties. Frequent changing of the medium removes unwanted hematopoietic stem cells, fats and macrophages. The plastic adherence ability of MSCs distinguishes them from hematopoietic cells. The morphological features of the cells were examined by an inverted microscope (Olympus CKX41), which confirmed the spindle-shaped fibroblasts and distinct colonies (Fig. 6A and B).

In order to further characterize the MSCs, some cell surface markers (CD34, CD44 and CD90 in this study) were investigated through flow cytometric analysis. Fig. 6C indicates that the majority of MSCs expressed positive surface markers for CD90 and CD44, but a lack of surface marker expression was observed for the hematopoietic stem cells CD34. According to this information, the cells isolated from bone marrow are mesenchymal.

**Fig. 7:**
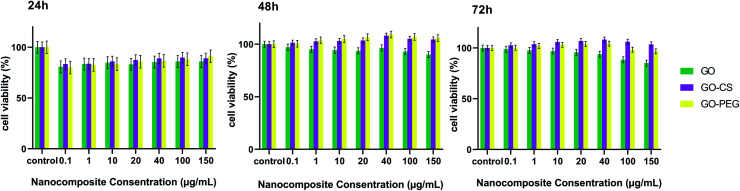
Cell viability of MSCs examined by an MTT assay for different concentrations of GO, GO–CS and GO–PEG after 24 h, 48 h and 72 h. Data indicate mean ± SD.

Ideal nanomaterials need to possess good biocompatibility for biomedical usage. Therefore the *in vitro* cytotoxicity of the prepared nanocomposites towards MSCs was investigated using an MTT assay. As shown in Fig. 7, cultured cells were treated with different concentrations (0.1, 1, 10, 20, 40, 100, 150 μg mL^−1^) of GO, GO–CS and GO–PEG in DMEM for 24, 48 and 72 hours. GO and its derivatives displayed no apparent cytotoxicity in 24 h and the cell viability was more than 80%. After incubation for 48 and 72 h, even at a high concentration, enhanced cell proliferation (nearly 105%) was observed. The cell survival level of the GO group remained a little lower (94%) than that of the GO–PEG and GO–CS groups (almost 107%). Oxygen functional groups on the GO surface can improve cell adhesion and viability through adsorbing proteins in the medium *via* covalent and non-covalent interactions. By modifying GO plates with CS and PEG, the GO surface was activated and cell growth easily increased. The results revealed that there were not only no significant cytotoxic effects for GO, GO–PEG and GO–CS, but also profitable cell growth for GO–PEG and GO–CS at 48 and 72 h, respectively. As many other studies have indicated, various factors, such as concentration, surface structure, size and shape, can affect the cytotoxicity of graphene-based materials.^49,55,56^

Animals were exposed to lateral spinal cord damage at T10 through compression. The treatment group received a sufficient amount of therapeutic nanocomposites (GO–PEG + GO–CS). Immediately after the SCI, the mice displayed no motor activity in their bilateral hind limbs, showing that the extreme SCI model was successful. After the operation, due to the lack of natural micturition reflexes, each mouse bladder had to be emptied manually every day until the mice were able to urinate again. This continued for one week. The mice also received antibiotics and normal saline regularly for five days.

**Fig. 8:**
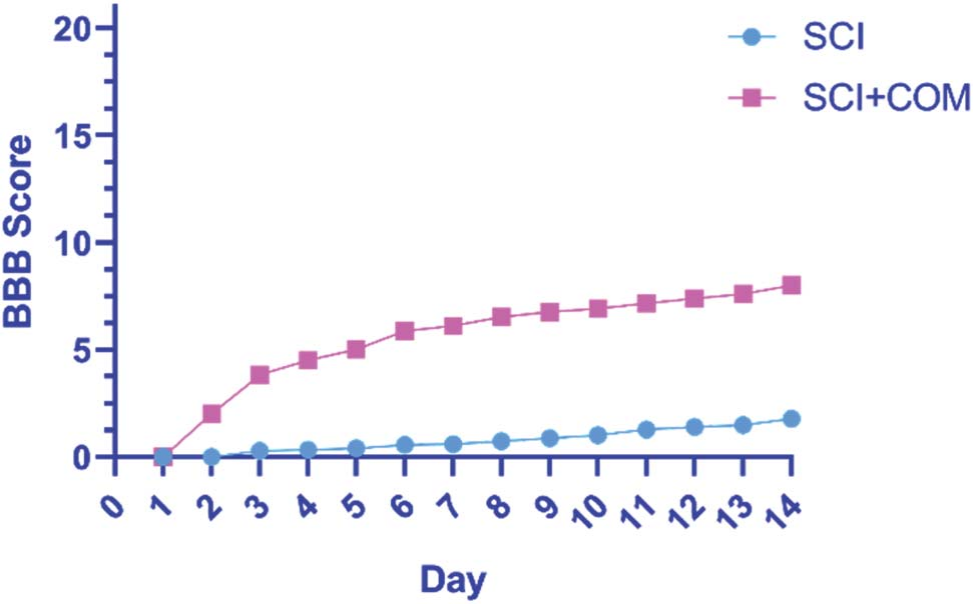
BBB open-field walking and functional recovery scores of mice hind limbs in the (SCI + COM) and (SCI) groups 1–14 days after injury (****p < 0.0001, determined by one-way ANOVA).

The primary functional outcomes were assessed according to the BBB locomotor scale for two weeks after surgery for a duration of 4 minutes. The 4 mice in the treatment group (SCI + COM) exhibited remarkable improvements on the BBB scale compared with the 4 mice in the control group (SCI). The mice in the treatment group achieved a mean locomotion score of 6 points (±standard error of the mean (SEM)) (Fig. 8). However, the mean of the control group was close to one, exhibiting a very limited increase. The results indicated that therapeutic nanocomposites, as a combination therapy, could effectively restore the activity in hind limbs after damage due to their intrinsic conductivity potential.

**Fig. 9:**
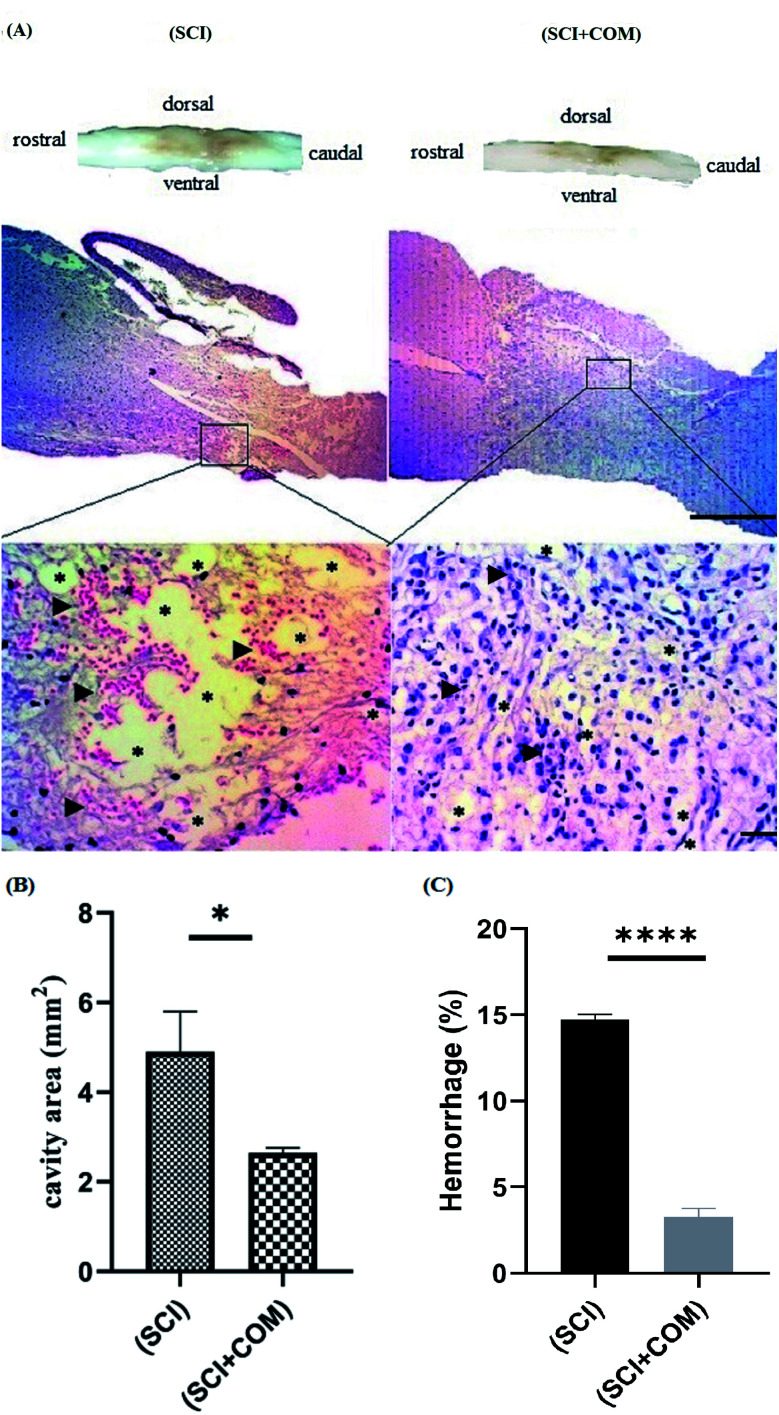
(A) Histological investigation of the spinal cord at 14 days post-injury by H&E staining at low magnification (4×), and high magnification (40×); higher magnification of the injury site obviously showed the cystic cavities (asterisks) and hemorrhage (arrowheads) in the (SCI) and (SCI + COM) groups. (B and C) Quantitative results for the cavity regions of sagittal segments and hemorrhage percentage at the lesion site in the spinal cord (*p < 0.05, ****p < 0.0001, *n* = 4 animals in each group). The scale bars are 1 mm and 50 μm.

H&E staining of longitudinal segments was used to evaluate the general histology of the (SCI) group and the (SCI + COM) group by light microscopy (Fig. 9A). The presence of several cystic cavities (asterisks), hemorrhage (arrowheads), edema, and necrosis was clearly seen around the lesion site in the (SCI) group. Formation of cavities (cysts) and scarring is a significant problem in the regeneration of adult mammalian spinal cords, as they interrupt the descending and ascending tracts and cause many unfavorable microenvironments.^57^ The specimens of the (SCI + COM) group displayed less prominent cavitation, hemorrhage, and necrosis because of the interaction of the anti-inflammatory and neuroprotective groups of the nanocomposites with the microenvironment of the lesions. Quantification of the cavity areas and hemorrhaging percentage indicated that there was a substantial decrease in the (SCI + COM) group compared with the (SCI) group (Fig. 9B and C). In this respect, a number of studies have shown the potential of GO and reduced GO in supporting SCI treatment. 2D and 3D GOx scaffolds were used to treat C6 spinal cord injury in rats. The obtained results confirmed that the oxygenated functional groups are likely to be responsible for specifically favoring higher protein adsorption and are particularly beneficial for fibrosis, inflammation, cell responses and atrophy.^8,58,59^ In a different study carried out with PEGylated graphene nanoribbons in L1 contusion SCI models, reduced numbers of astrocytes, improved locomotor function and regenerated axons were observed after 5 weeks.^60^ In our case, it can be found that the presence of CS and PEG on GO plates not only reconnects the pathways and increases locomotive activity rapidly but also inhibits the acute inflammatory response, edema, hemorrhage and glial scar formation. More detailed tissue staining, such as immunohistochemistry, will be explored in later studies.

## Conclusion

SCI is one of the most crippling human pathologies, with a profound effect on quality of life and on civilization as a whole. Unfortunately, due to the varying causes of degeneration and suppression of development following injuries, there is no permanent treatment for this type of disease. Important developments in the production of bio-nanomaterials for SCI repair have been reported over the last decade and the combination strategy will be more effective in this application.

Nano-sized graphene oxide composites (GO–CS, GO–PEG) as biomaterials have been successfully synthesized in this work. Following physicochemical characterization, we analyzed the *in vitro* MSC toxicity and confirmed that the prepared nanocomposites were non-toxic with a positive impact (∼10 percent) on cell growth and proliferation through the interaction of oxygen-containing units with the proteins in the medium. We inserted a combination of nanocomposites into the spinal cords of mice with T10 injuries. Investigation of the functional regeneration and subacute tissue reactions in the injured spinal cords of the mice showed evidence that these frameworks promote tissue repair as soon as two weeks after spinal cord injury and prevent growth of the lesion in the absence of drugs and/or growth factor. A deep understanding of how embedded nanocomposites interact with host tissues needs to be defined. Applying a wide range of protective and inducing drugs, growth factors and cells combined with these materials could enhance functional recovery and regrowth of neurites (axons and neurons) with reduced glial scarring. The authors strongly recommend the development of materials to monitor the state of the lesion site and the localization of cells, and to detect regeneration mechanisms.

## Conflicts of interest

The authors have no financial conflict to declare.

## Supplementary Material
